# A hierarchical regulatory network analysis of the vitamin D induced transcriptome reveals novel regulators and complete VDR dependency in monocytes

**DOI:** 10.1038/s41598-021-86032-5

**Published:** 2021-03-22

**Authors:** Timothy Warwick, Marcel H. Schulz, Stefan Günther, Ralf Gilsbach, Antonio Neme, Carsten Carlberg, Ralf P. Brandes, Sabine Seuter

**Affiliations:** 1 Institute for Cardiovascular Physiology, Medical Faculty, Goethe University, Theodor-Stern-Kai 7, 60590 Frankfurt am Main, Germany; 2 Institute for Cardiovascular Regeneration, Goethe University, Frankfurt am Main, Germany; 3 German Center for Cardiovascular Research (DZHK), Partner site Rhein-Main, 60590 Frankfurt am Main, Germany; 4 Max-Planck-Institute for Heart and Lung Research, Bad Nauheim, Germany; 5 School of Medicine, Institute of Biomedicine, University of Eastern Finland, 70211 Kuopio, Finland; 6 Institute for Applied Mathematics, Merida Research Unit, National Autonomous University of Mexico, C.P. 97302 Sierra Papacal Merida, Yucatan Mexico

**Keywords:** Epigenetics, Gene expression, Gene regulation, Sequencing, Endocrinology

## Abstract

The transcription factor vitamin D receptor (VDR) is the high affinity nuclear target of the biologically active form of vitamin D_3_ (1,25(OH)_2_D_3_). In order to identify pure genomic transcriptional effects of 1,25(OH)_2_D_3_, we used VDR cistrome, transcriptome and open chromatin data, obtained from the human monocytic cell line THP-1, for a novel hierarchical analysis applying three bioinformatics approaches. We predicted 75.6% of all early 1,25(OH)_2_D_3_-responding (2.5 or 4 h) and 57.4% of the late differentially expressed genes (24 h) to be primary VDR target genes. *VDR* knockout led to a complete loss of 1,25(OH)_2_D_3_–induced genome-wide gene regulation. Thus, there was no indication of any VDR-independent non-genomic actions of 1,25(OH)_2_D_3_ modulating its transcriptional response. Among the predicted primary VDR target genes, 47 were coding for transcription factors and thus may mediate secondary 1,25(OH)_2_D_3_ responses. CEBPA and ETS1 ChIP-seq data and RNA-seq following *CEBPA* knockdown were used to validate the predicted regulation of secondary vitamin D target genes by both transcription factors. In conclusion, a directional network containing 47 partly novel primary VDR target transcription factors describes secondary responses in a highly complex vitamin D signaling cascade. The central transcription factor VDR is indispensable for all transcriptome-wide effects of the nuclear hormone.

## Introduction

The lipophilic molecule 1α,25-dihydroxyvitamin D_3_ (1,25(OH)_2_D_3_) is the most active metabolite of the biologically inert micronutrient vitamin D_3_. The endocrine hormone 1,25(OH)_2_D_3_ binds with high affinity to the transcription factor vitamin D receptor (VDR), a member of the nuclear receptor superfamily^1^. Although particularly highly expressed in the small intestine and the kidney, VDR is also found in immune cells and many other organs, albeit at lower level^2^. This explains why VDR not only plays a role in the regulation of calcium and phosphorus homeostasis^3^, but also modulates other physiological functions, including innate and adaptive immune responses^4^. According to the canonical nuclear receptor signaling model, VDR binds as a heterodimer with the nuclear receptor retinoid X receptor (RXR) to response elements formed by a direct repeat of two hexameric sequence motifs spaced by three nucleotides (DR3)^5,6^. Numerous transcriptomic and epigenome-wide data obtained in human monocytic THP-1 cells were used to refine this model to better describe genomic vitamin D signaling involving possible modulation of the 3-dimensional chromatin structure as well as the action of pioneer factors like purine-rich box 1 (PU.1 or SPI1) and CCAAT/enhancer binding protein α (CEBPA)^7–17^.

In addition to the genomic actions, rapid non-genomic effects have also been described in response to 1,25(OH)_2_D_3_ which are independent of transcriptional regulation. These effects appear to be cell type-specific and are mediated by kinases such as phospholipase A2 (PLA2), phospholipase C (PLC), protein kinase A (PKA), protein kinase C (PKC), Ca^2+^/calmodulin-dependent protein kinase (PKCaMII), mitogen-activated protein kinases (MAPK), Src tyrosine phosphorylation and phosphatidylinositol 3-kinase (PI3K)^18–25^. Reported physiological effects of non-genomic responses to 1,25(OH)_2_D_3_ comprise modulations of calcium uptake and secretion, intracellular pH, insulin secretion and cell migration, but their overall significance in vivo is unclear^18,21,24–30^. There is some controversy in the identification of the receptor(s) which convey 1,25(OH)_2_D_3-_induced non-genomic cellular function. Some reports state a completely VDR-independent propagation via a novel membrane receptor^18,31–34^. VDR has been found localized in membrane caveolae and to interact or synergize with the membrane receptor, Protein disulphide isomers family A member 3 (PDIA3, also called 1,25D3-MARRS or ERp57)^35–39^. It has also been reported that 1,25(OH)_2_D_3_ can induce non-genomic effects by binding to an alternative ligand binding pocket in the classical nuclear VDR^28,29^. Thus, there may be different parallel mechanisms for non-genomic actions of 1,25(OH)_2_D_3_, for some of which both VDR and PDIA3 are required^20,36,40–43^. It is generally agreed that non-genomic 1,25(OH)_2_D_3_ signaling facilitates its genomic regulation, for example by activating RXR or other transcription factors by phosphorylation^41,44^. In this study, we knocked down the VDR to determine whether any transcriptome-wide effects of 1,25(OH)_2_D_3_ can be observed in the absence of its nuclear receptor.

Further, we used the EpiRegio database of regulatory elements (REMs)^45^ and the TEPIC 2 framework for transcription factor binding site prediction^46^ to study primary and secondary effects of vitamin D.

## Material and methods

### Cell culture

The human acute monocytic leukemia cell line THP-1^47^ is a well responding and physiologically meaningful model system for the investigation of 1,25(OH)_2_D_3_-triggered physiological processes, such as innate immunity and cellular growth^8,48–50^. The cells were grown in RPMI 1640 medium, supplemented with 10% fetal calf serum (FCS), 2 mM L-glutamine, 0.1 mg/ml streptomycin and 100 U/ml penicillin, and were kept at 37 °C in a humidified 95% air/5% CO_2_ incubator. Prior to mRNA or chromatin extraction, cells were grown overnight in phenol red-free medium supplemented with charcoal-stripped FCS and then treated with vehicle (0.1% ethanol (EtOH)) or 100 nM 1,25(OH)_2_D_3_ (Sigma-Aldrich).

Lenti-X 293 T cells (Takara Bio) were grown in DMEM medium supplemented with 10% FCS, 0.1 mg/ml streptomycin and 100 U/ml penicillin and were kept at 37 °C in a humidified 95% air/5% CO_2_ incubator.

### CRISPR/Cas9-mediated knockout of VDR

THP-1 cells stably expressing type II CRISPR RNA-guided endonuclease Cas9 (Cas9), generated by lentiviral transduction with the plasmid lentiCas9-Blast (Addgene #52,962) into THP-1 cells from DSMZ (ACC16), and the sequences for the non-targeting control guide RNAs (gRNAs) were kindly provided by Dr. Frank Schnuetgen (Department of Molecular Hematology, Goethe University, Frankfurt/Main, Germany).

Specific gRNAs targeting the *VDR* gene have been designed using the Benchling life sciences R&D cloud^51^ and CHOPCHOP^52^. Sense and antisense oligonucleotides containing the 19 bp gRNA sequence plus the required overhangs matching the *BsmBI*-cut vector were ordered (Sigma-Aldrich) and annealed (Supplementary Table S1). Then, *BsmBI* (*Esp3I*) (Thermo Fisher) and T4 DNA ligase (Thermo Fisher) were used to clone the annealed gRNAs into the plasmid LentiGuide-Puro (Addgene #52,963) with the Golden Gate assembly protocol^53^. Correct insertion of the gRNAs into the plasmid was verified by Sanger sequencing.

Cell-free, lentiviral supernatants were produced by polyethylenimine (PEI)-based transient co-transfection of Lenti-X 293 T cells. Briefly, the pLentiGuide-Puro-gRNA vector, the lentiviral gag/pol packaging plasmid psPAX (Addgene #12,260) and the envelope plasmid encoding the glycoprotein of vesicular stomatitis virus (VSV-G) (pMD2.G, Addgene #12,259) were transfected at a molar ratio of 3:1:1 by standard PEI transfection. 24 h and 48 h post transfection, two consecutive viral supernatants were harvested, cleared through 0.45-µm pore-size PVDF membrane filter (Millipore), combined and stored at − 80 °C.

Prior to lentiviral transduction, the THP-1/Lenti-Cas9-Blast cells were re-selected with Blasticidine S hydrochloride (50 µg/ml) for 3 days. The transduction with the gRNA-expressing plasmids was performed in two consecutive spinoculations. Briefly, 0.2 × 10^6^ cells were pelleted and resuspended in 1 ml of the lentiviral particles. Polybrene (8 µg/ml) was added and the cells were centrifuged for 90 min with 1000 × g at room temperature. The supernatant was discarded, the cells resuspended in 1 ml full growth medium and incubated for 48–72 h. Then, the second spinoculation was carried out with 800 µl of lentivirus. After another 48–72 h incubation, puromycin (2 µg/ml) was added to select the transduced cells for 10–14 days.

Prior to each experiment for RNA extraction, the *VDR* knockout cells were re-selected with puromycin (2 µg/ml) for three days.

### Western blot

Cells were washed twice with phosphate-buffered saline (PBS) and lysed with Triton X-100 lysis buffer (20 mM TRIS–HCl pH 7.0, 150 mM NaCl, 10 mM sodium pyrophosphate, 20 mM NaF, 1% Triton X-100, 2 mM orthovanadate, 10 nM okadaic acid, protein inhibitor mix (2 ng/ml of antipain, aprotinin, chymostatin, leupeptin, pepstatin and trypsin inhibitor, each), 40 μg/ml phenylmethylsulfonyl fluoride). Cells were centrifuged for 10 min at full speed at 4 °C. The supernatant was transferred and after determination of protein concentration by the Bradford assay, equal amounts of protein were boiled in sample buffer and separated by SDS-PAGE (8%). The gels were blotted onto a nitrocellulose membrane and blocked in Rotiblock (Carl Roth). The anti-VDR antibody (Cell Signaling Technology, #12,550) and the β-actin antibody (Cell Signaling Technology, #4970S) were used in a 1:1000 dilution. The blots were developed with infrared-fluorescent-dye-conjugated secondary antibodies (LI-COR Biosciences) and detected with an Odyssey Classic system (LI-COR Biosciences).

### RNA isolation

Total RNA was extracted using the Quick-RNA Miniprep Kit (Zymo Research) according to the manufacturer’s instructions. RNA quality was assessed on a Fragment Analyzer (Agilent). Four independent replicate experiments (biological repeats) were performed for RNA sequencing (RNA-seq).

### RNA-seq

RNA-seq libraries were prepared from 1 µg total RNA using the VAHTS Stranded mRNA-seq Library Prep Kit for Illumina V2 (Vazyme, #NR612) including poly(A) enrichment. Single end sequencing was conducted to 75 bp read length on the NextSeq500 platform using standard Illumina protocols resulting in roughly 20 M reads per library. The resulting raw reads were assessed for quality, adapter content and duplication rates with FastQC. Then, the high-quality reads were aligned to the reference genome (hg19) using Spliced Transcripts Alignment to a Reference (STAR) with the parameter –quantMode GeneCounts for gene-level quantification and otherwise default parameters^54^. Differential gene expression was computed using DESeq2, which implements a negative binomial test over the reads in each two conditions (1,25(OH)_2_D_3_-treated/solvent-treated or NTC control/*VDR* knockout), with standard parameters and an adjusted p-value cutoff of 0.05^55^. Information regarding which batch of THP-1 cells each sample originated from was provided to DESeq2 to correct for batch effects.

### ETS1 ChIP-seq

ChIP assays were performed as described by Zhang et al*.*^56^ with some modifications. After treatment of 20 × 10^6^ THP-1 cells with 100 nM 1,25(OH)_2_D_3_ for 24 h, nuclear proteins were cross-linked to genomic DNA by adding formaldehyde directly to the medium to a final concentration of 1% and incubating at room temperature for 10 min on a rocking platform. Cross-linking was stopped by adding glycine to a final concentration of 0.125 M and incubating at room temperature for 10 min on a rocking platform. The cells were collected by centrifugation and washed twice with ice cold PBS. The cell pellets were subsequently resuspended twice in 10 ml cell lysis buffer (0.1% SDS, 1 mM EDTA, 150 mM NaCl, 1% Triton X-100, 0.1% sodium deoxycholate, protease inhibitors, 50 mM HEPES–KOH, pH 7.5) and once in 10 ml nuclear lysis buffer (1% SDS, 1 mM EDTA, 150 mM NaCl, 1% Triton X-100, 0.1% sodium deoxycholate, protease inhibitors, 50 mM HEPES–KOH, pH 7.5). After two washes with cell lysis buffer, the chromatin pellet was resuspended in 700 µl of SDS lysis buffer (1% SDS, 10 mM EDTA, protease inhibitors, 50 mM Tris–HCl, pH 8.1) and the lysates were sonicated in a Bioruptor Plus (Diagenode) to result in DNA fragments of 200 to 500 bp. Cellular debris was removed by centrifugation. 340 µl aliquots of the lysate were diluted 1:5 in IP dilution buffer (1% Triton X-100, 2 mM EDTA, 150 mM NaCl, protease inhibitors, 20 mM Tris–HCl, pH 7.5). 4 µg anti-ETS1 antibody (Santa Cruz, sc-350X), previously used by the ENCODE project (data available at Gene Expression Omnibus (GEO)^57^ at GSE32465) were coated to 60 µl Dynabeads Protein G (Invitrogen) in an overnight incubation at 4 °C. The pre-formed bead-antibody complexes were then washed twice with beads wash buffer (0.1% Triton X-100, PBS, protease inhibitors) and added to the chromatin aliquots. The samples were incubated for overnight at 4 °C on a rotating wheel to form and collect the immuno-complexes. The beads were washed sequentially for 5 min on a rotating wheel with 1 ml of the following buffers, each: twice cell lysis buffer, once high salt buffer (0.1% SDS, 1% Triton X-100, 1 mM EDTA, 350 mM NaCl, 0.1% sodium deoxycholate, 50 mM HEPES–KOH, pH 7.5), once ChIP wash buffer (250 mM LiCl, 1% Nonidet P-40, 0.5% sodium deoxycholate, 1 mM EDTA, 10 mM Tris–HCl, pH 8.0) and twice 1 ml TE buffer (1 mM EDTA, 10 mM Tris–HCl, pH 8.0). Then, the immune complexes were eluted using 250 µl ChIP elution buffer (1% SDS, 10 mM EDTA, 50 mM Tris–HCl, pH 7.5) at 37 °C for 30 min with rotation. The elution was repeated with a 10 min rotation and the supernatants were combined. The immune complexes were reverse cross-linked at 50 °C for 2 h in the presence of proteinase K (Fermentas) in a final concentration of 40 µg/ml. The genomic DNA was isolated with the ChIP DNA Clean&Concentrator Kit (Zymo Research). Two biological replicates have been prepared for the ETS1 sequencing (ChIP-seq).

2–10 ng of each ChIP DNA template was used for library preparation with the NEBNext Ultra II kit (New England Biolabs) and libraries were sequenced at 50 bp read length on a HiSeq2000 system using standard Illumina protocols at the Gene Core of the EMBL (Heidelberg, Germany). ChIP-seq data was aligned with the human reference genome version hg19 using Bowtie software version 1.1.1 with the following essential command line arguments: bowtie -n 1 -m 1 -k 1 -e 70 –best. The aligned input and ETS1 reads were converted to sorted BAM format using samtools and, after merging the read sets per sample, converted to TDF format using igvtools, in order to allow efficient visualization in the Integrative Genomics Viewer (IGV) genome browser. Statistically significant ChIP-seq peaks were identified using Model-based Analysis of ChIP-Seq data (MACS) version 2^58^ with the following essential command line arguments: macs2 callpeak –bw 150 –keep-dup 1 –g hs –qvalue = 0.01 –m 5 50 –bdg. Peaks are declared when enriched regions, compared with the corresponding loci in the input, are found. Otherwise, default parameters were used. ETS1 ChIP-seq raw data are available at GEO at GSE157209.

### Previously published data

VDR ChIP-seq data in THP-1 cells is publicly available at NCBI GEO under the accession GSE89431, originally published by Neme et al*.*^11^. CEBPA ChIP-seq data can be accessed under GSE119556^13^ and H3K27ac ChIP-seq under GSE107851^12^. Formaldehyde-assisted isolation of regulatory elements sequencing (FAIRE-seq) data in THP-1 cells after 24 h of 100 nM 1,25(OH)_2_D_3_ treatment is available from the same source under the accessions GSE69297 and GSE69303, originally published by Seuter et al*.*^15^. RNA-seq time course data in THP-1 cells with 2.5 h, 4 h and 24 h of 100 nM 1,25(OH)_2_D_3_ treatment is accessible at the accession GSE69303^11,15^.

### Identification of direct VDR target genes

TEPIC (version 2.2) is a universal software for the annotation of genes and integration of epigenomic, ChIP-seq and TF motif data^46^. In order to make use of the VDR ChIP-seq data to identify direct VDR target genes, we used (i) TEPIC 2 to associate VDR ChIP-seq peaks to genes by considering a window of 50 kb centered at the transcription start site (TSS) of genes. Further, (ii) we overlapped VDR ChIP-seq peaks with annotated REMs of the EpiRegio database^45^. REMs in EpiRegio are linked to genes. Each gene that had at least one REM that was linked to it and overlapped a VDR ChIP-seq peak was considered a direct VDR target.

Finally, (iii) we made use of FAIRE-seq data in THP-1 cells after 1,25(OH)_2_D_3_ treatment and looked for predicted VDR binding sites using position weight matrices of VDR or RXRA::VDR obtained from merged JASPAR 2020 (MA0693.2, MA0074.1)^59^, HOCOMOCO (VDR_HUMAN.H11MO.1.A, VDR_HUMAN.H11MO.0.A)^60^ and Kellis-ENCODE^61^ motif sets included in TEPIC 2.2. Transcription factor binding affinities to regions of open chromatin are calculated using TRAP. Predicted VDR sites were assigned to genes if they were present in a window of 50 kb around the TSS of a gene. Only affinities meeting a threshold determined by user-supplied random sequences were used to assign transcription factors to genes. Calculated affinities are subject to exponential decay depending on the distance of the predicted transcription factor binding site from the TSS of the respective gene.

The direct target genes obtained by the three approaches above were intersected with sets of DEGs after 1,25(OH)_2_D_3_ treatment measured with RNA-seq.

### Prediction of secondary transcription factor regulation

We used TEPIC 2.2 to annotate open-chromatin regions detected with FAIRE-seq in THP-1 cells after 24 h of 1,25(OH)_2_D_3_ treatment. Transcription factor binding sites were predicted using position specific energy matrices derived from JASPAR 2020^59^, HOmo sapiens COmprehensive MOdel COllection (HOCOMOCO)^60^ and Kellis ENCODE^61^ motif databases, which are part of the TEPIC 2 repository.

The DYNAMITE workflow of TEPIC 2, was used to learn the importance of individual transcription factors to gene expression changes of the subset of DEGs in THP-1 cells after 24 h of 100 nM 1,25(OH)_2_D_3_ treatment, which were not identified as direct VDR target genes. DYNAMITE used transcription factor-gene scores calculated by TEPIC 2 from the FAIRE-seq data before and after treatment for a set of 720 transcription factors. These scores were used as features for a sparse logistic regression classifier that predicts up- and down-regulated DEGs. The classifier learns a coefficient for each transcription factor included in the analysis, which describes the importance of the respective transcription factor to the modulation of gene expression in the dataset. We normalized the coefficients obtained from the classifier to the range [0,1].

### Transcription factor network construction

The transcription factor network was constructed using the *igraph* package in R (version 4.0.0). Edges were added between VDR and transcription factors predicted to be primary 1,25(OH)_2_D_3_ targets. Further edges were added between early-induced transcription factors and the rest of the induced transcription factors, if the early-induced transcription factor was predicted to bind at the gene locus (TSS + /- 25 kb) of the second transcription factor at an affinity greater than a given threshold by TEPIC 2. The network was plotted using the *ggraph* package in R.

### Pathway enrichment analysis

Pathway enrichment analysis was performed using the R (4.0.2) package ReactomePA (1.32.0)^62^. Significantly overrepresented pathways were those with a Benjamini–Hochberg adjusted P < 0.05.

## Results

### The majority of 1,25(OH)_2_D_3_ responsive genes are primary VDR targets

In THP-1 cells treated with 1,25(OH)_2_D_3_ for 2.5, 4 or 24 h, the total number of DEGs had been reported by Neme et al*.*^11^ as 3650. Among these were 19 non-annotated transcripts, which were excluded for this study. Of the remaining 3631 annotated genes, 513 genes were differentially expressed after 2.5 or 4 h of stimulation and as such were classified as early 1,25(OH)_2_D_3_-responsive genes. No genes were transiently differentially expressed. Commonly, early responding genes are considered directly regulated by a transcription factor. Thus, in our previous studies in THP-1 cells, we have classified genes as primary vitamin D targets if they were differentially expressed at early time points of 1,25(OH)_2_D_3_ treatment, whereas those differentially expressed only at a late time point (24 h) were classified as secondary target genes^11,63^. According to this classification, the vast majority of all differentially expressed genes (DEGs) were secondary vitamin D target genes (3,133), while only 517 genes were early responding primary targets. However, since there are many VDR-bound regions (ChIP-seq) close to many of these so-called secondary vitamin D target genes, it seems likely that these genes can be regulated by VDR-bound enhancer elements. Hence, a better method to identify direct transcription factor targets was needed.

**Figure 1:**
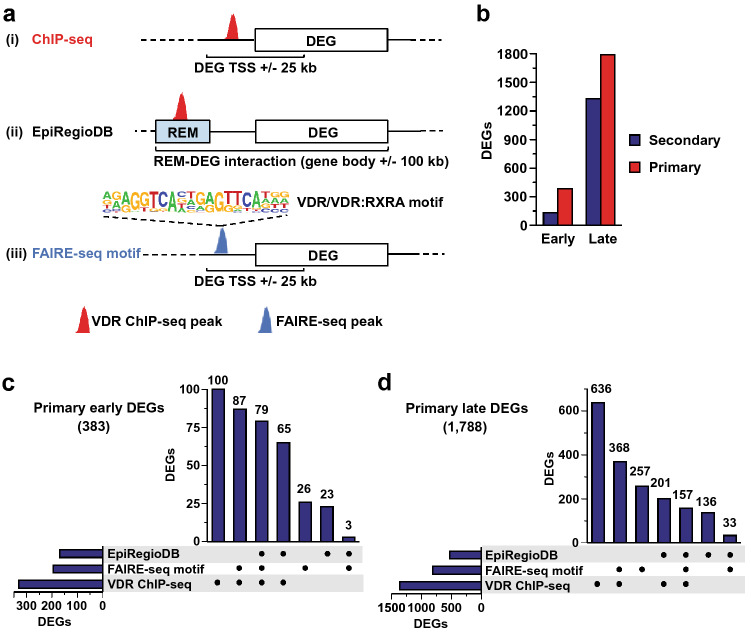
Identification of direct VDR target genes. Three different approaches have been used to predict primary vitamin D target genes from early or late 1,25(OH)_2_D_3_-responding genes measured with RNA-seq^11^ (**a**). The first two identified direct target genes using VDR ChIP-seq peaks that were (i) located in a window of ± 25 kb around the TSS of genes or ii) overlapping a regulatory element (REM) in the EpiRegio database that was linked to a DEG. The third (iii) used FAIRE-seq peaks with a predicted VDR binding motif that are located in a window of ± 25 kb around the TSS of DEGs (**b**). The proportions of primary vitamin D target genes predicted by at least one of the three methods are shown for both early and late DEGs. The numbers of primary VDR target genes predicted by one, two, or all three of the approaches are depicted in upset plots for early (2.5 or 4 h; **c**) and late (24 h; **d**) DEGs after 100 nM 1,25(OH)_2_D_3_ treatment of THP-1 cells^11,15^.

However, linking regulatory regions to target genes is not easy and each method has its limitations^64,65^. Thus, in the present study, we combined three different approaches to predict primary vitamin D target genes from 1,25(OH)_2_D_3_-responding genes (Fig. 1a) to overcome limitations of an individual approach. In the first approach (i), TEPIC 2 was used to search for VDR ChIP-seq peaks in a window of ± 25 kb around the TSS of genes differentially expressed by the 1,25(OH)_2_D_3_ treatment. The second approach (ii) used REMs linked to genes from the EpiRegio database^45^. EpiRegio holds elements which have been identified by correlations between chromatin accessibility and gene expression at loci in a window of gene body ± 100 kb using data from the BLUEPRINT^66^ and the Roadmap consortia^67^. VDR ChIP-seq peaks overlapping with an REM from EpiRegio were linked to DEGs^45^. In the third approach (iii), the TEPIC 2 framework was used to search for VDR binding motifs obtained from diverse motif databases (see Methods) overlapping FAIRE-seq peaks that were located in a window of ± 25 kb around the TSS of 1,25(OH)_2_D_3_ DEGs. TEPIC 2 is a method that computes transcription factor gene scores based on position specific energy matrices of transcription factors and the signal of an open-chromatin assay^46^. By limiting the search space around genes to nearby regions of open chromatin, the prediction of contextually important transcription factor binding is more likely than using a continuous search space of the gene region without epigenetic information.

According to the primary VDR target gene criteria set out above, 383 early-induced 1,25(OH)_2_D_3_-responsive genes (75.6%) were predicted to be primary VDR targets by any of the three methods. The remaining 130 genes (24.4%) were classified as non-primary (Fig. 1b). Amongst these early primary targets were classical 1,25(OH)_2_D_3_-responsive genes including cathelicidin antimicrobial peptide (*CAMP*), heparin binding EGF like growth factor (*HBEGF*) and ninjurin 1 (*NINJ1)*^8,68,69^.

There were 3,117 genes differentially expressed after 24 h of 1,25(OH)_2_D_3_ treatment, which were not differentially expressed at either 2.5 h or 4 h, and as such were classified as late 1,25(OH)_2_D_3_-responsive genes. Of these late-responding genes, 1,788 (57.4%) were classified as primary VDR targets by the aforementioned criteria, leaving 1,329 (42.6%) of the late-responding 1,25(OH)_2_D_3_-regulated genes as secondary vitamin D target genes (Fig. 1c).

Of the early induced vitamin D target genes, 61.1% (234 of 383) of the primary VDR target gene predictions have been confirmed by at least two of the three approaches, 79 genes (20.6%) by all three. 100 genes (26.1%) were assigned primary early VDR targets only based on window-based VDR binding (Fig. 1c).

Of the late primary 1,25(OH)_2_D_3_–responding genes, 8.8% (157 of 1,788) were predicted to be primary VDR target genes by all three approaches, and 42.4% (759 of 1,788) by at least two of the three methods. There were 368 genes (20.6%) assigned as primary VDR targets by the combination of transcription factor binding site prediction using TEPIC 2 in conjunction with 1,25(OH)_2_D_3_-stimulated FAIRE-seq data and a window based VDR peak to gene assignment and another 636 (35.6%) by the VDR association alone (Fig. 1d). At the early time points, almost half of the genes predicted to be subject to regulation by REMs taken from the EpiRegio database were also predicted to be primary VDR targets by window-based VDR ChIP-seq peak assignment and transcription factor binding site prediction by TEPIC 2 using FAIRE-seq data (79 of 170, 46.5%), while this ratio was only 29.8% (157 of 527) for the late 1,25(OH)_2_D_3_–responding genes (Figs. 1c and d).

Taken together, using three bioinformatics approaches including VDR ChIP-seq data, VDR motif search in open chromatin regions and REMs identified from multiple published genome-wide datasets, 75.6% of all early 1,25(OH)_2_D_3_-responding and 57.4% of the late DEGs were predicted to be primary VDR target genes.

### Transcriptome-wide effects of 1,25(OH)_2_D_3_ are entirely VDR dependent

THP-1 VDR knockout cells were created in order to determine the contribution of VDR-dependent and independent mechanisms to 1,25(OH)_2_D_3_-regulated gene expression. The knockout was achieved by lentiviral clustered regularly interspaced short palindromic repeats (CRISPR)/Cas9-mediated gene editing. Sufficient knockout on protein level was confirmed by Western blot for knockout lines generated with four different gRNAs (Supplementary Figure S1, Supplementary Table S1). Quadruplicate RNA-seq experiments from VDR knockout cells (line L56) and non-targeting gRNA control cells (NTC2, L52) were performed after treatment with 100 nM 1,25(OH)_2_D_3_ or solvent for 4 or 24 h. The numbers of genes differentially expressed after 4 and 24 h stimulation with 1,25(OH)_2_D_3_ were 109 and 1,341, respectively (Supplementary Table S2). This were fewer genes than were identified in our previous RNA-seq with THP-1 wildtype cells^11^. However, similarly as in the previously published dataset from wildtype THP-1 cells, there was only one gene transiently early regulated (Supplementary Figure S2a) and the overlap between the DEGs identified from wildtype and the CRISPR/Cas9 control cells is high (Supplementary Figures S2b and c).

**Figure 2:**
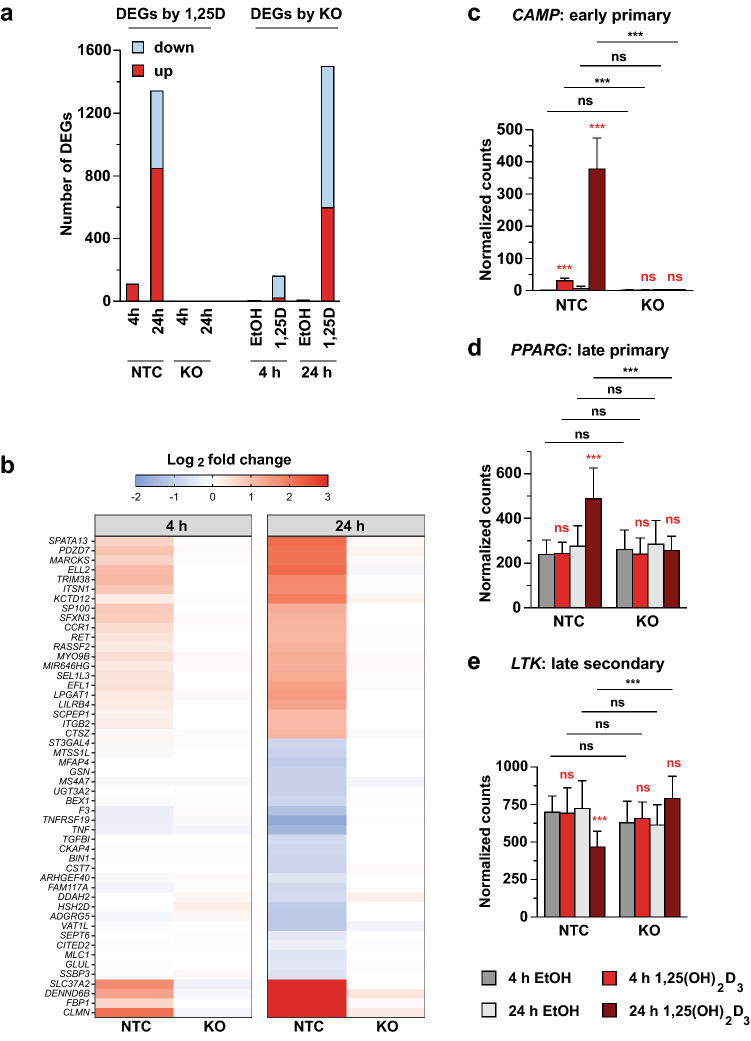
VDR knockout abolishes transcriptomic vitamin D response. The VDR was knocked out by CRISPR/Cas9 genome editing in THP-1 cells. VDR knockout (KO) cells and control (NTC) cells were treated with 1,25(OH)_2_D_3_ or solvent (EtOH) for 4 or 24 h and four biological repeats of RNA-seq have been performed. The numbers of DEGs by either the 1,25(OH)_2_D_3_ stimulation or the VDR knockout are displayed (**a**). The log2 fold changes in gene expression of the top 50 most significant DEGs in both VDR KO and NTC control cells demonstrates the almost complete loss of 1,25(OH)_2_D_3_ inducibility (**b**). There rarely is any effect of the VDR KO on the basal expression levels of vitamin D target genes, which is shown for some exemplary genes (**c–e**).

As in the wildtype cell dataset, just over half of all identified DEGs are upregulated in response to 1,25(OH)_2_D_3_ exposure (Fig. 2a, left). Importantly, in *VDR* knockout cells, not a single gene could be detected to be differentially expressed by 1,25(OH)_2_D_3_. Three observations from the differential expression analysis between VDR knockout and control cells confirmed the complete dependence of 1,25(OH)_2_D_3_ regulation on the presence of VDR: (i) in ligand-treated cells similar numbers of genes were differentially expressed as after stimulation with 1,25(OH)_2_D_3_ in control cells (Fig. 2a, right), (ii) the overlap between the genes differentially expressed by the VDR knockout at the two time points is similarly high as for the differential expression by 1,25(OH)_2_D_3_ (Supplementary Figures S2d and a), and (iii) there is a high intersection ratio between 1,25(OH)_2_D_3_-differential genes in control cells and knockout-differential genes in 1,25(OH)_2_D_3_-stimulated cells for both time points (Supplementary Figure S3a and b). In solvent-treated cells, hardly any gene was significantly affected by the VDR knockout, indicating that VDR depletion only affects vitamin D target genes (Fig. 2a, right). The heatmap displaying the changes in gene expression by 1,25(OH)_2_D_3_ (log2 scale) demonstrates that for most genes the effect of 1,25(OH)_2_D_3_ on mRNA expression is not only reduced but completely lost upon VDR depletion (Fig. 2b). This is illustrated by the representation of the normalized counts for the early primary vitamin D target genes *CAMP*, ZFP36 ring finger protein (*ZFP36*) and potassium voltage-gated channel modifier subfamily F member 1 (*KCNF1*), the late primary VDR targets peroxisome proliferator activated receptor gamma (*PPARG*) and C-X-C motif chemokine ligand 8 (*CXCL8,* encoding interleukin 8 (IL8)) and the late secondary target leukocyte receptor tyrosine kinase (*LTK*) (Figs. 2c-e and S4a-c).

In summary, VDR knockout led to a complete loss of 1,25(OH)_2_D_3_–induced gene regulation in monocytic THP-1 cells, *i.e.*, transcriptome-wide effects of vitamin D were entirely mediated by VDR.

### 1,25(OH)_2_D_3_ regulates transcription factor mRNA expression

**Figure 3:**
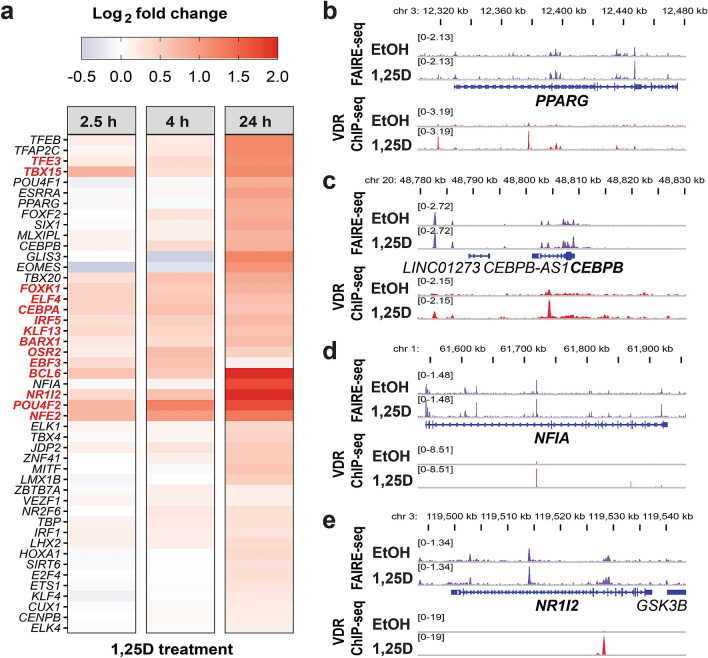
Identification of transcription factors that mediate secondary vitamin D responses. Log2 fold changes in gene expression versus time-matched control samples are displayed per time point for 47 genes coding for transcription factors, which have been predicted to be primary 1,25(OH)_2_D_3_ targets genes (**a**). Display of VDR ChIP-seq^11^ and FAIRE-seq^15^ data in the IGV browser illustrates binding of VDR in regions of open chromatin in the loci of the four transcription factors *PPARG*, *CEBPB*, *NFIA* and *NR1I2* (**b–e**).

Of the in total 3,631 DEGs, 2,114 (58.2%) were predicted to be primary VDR targets according to the criteria and methods used in this study. For the physiological impact of vitamin D signaling, secondary responses are as important as primary effects. Previously, the four transcription factors B-cell CLL/lymphoma 6 (BCL6), nuclear factor erythroid 2 (NFE2), E74-like ETS transcription factor 4 (ELF4) and POU class 4 homeobox 2 (POU4F2) have been shown to mediate secondary effects of vitamin D^70^. To globally identify the indirect mechanisms by which the 1,517 non-primary vitamin D target genes (41.8%) were regulated, we screened the primary VDR target genes for transcription factor genes and identified 47 among them. A subset of these genes were early vitamin D target genes, including the pioneer factors *CEBPA* and ETS proto-oncogene 1, transcription factor (*ETS1*) as well as *BCL6* and *NFE2*. The remaining 34 transcription factor genes only showed upregulation after 24 h of 1,25(OH)_2_D_3_ treatment. Among these were genes such as nuclear factor I A (*NFIA*)*,* the nuclear receptor *PPARG* and cut like homeobox 1 (*CUX1*) (Fig. 3a). VDR associated to the gene loci of *PPARG*, *CEBPB*, *NFIA* and *NR1I2* (coding for the pregnane X receptor (PXR)) in regions of open chromatin as determined by VDR ChIP-seq and FAIRE-seq, respectively. This supported the prediction that these genes are direct VDR targets (Fig. 3b-e).

Taken together, among the genes differentially expressed by 1,25(OH)_2_D_3_ and being predicted to be primary VDR targets 47 were coding for transcription factors.

### Characterization of secondary 1,25(OH)_2_D_3_ responses

**Figure 4:**
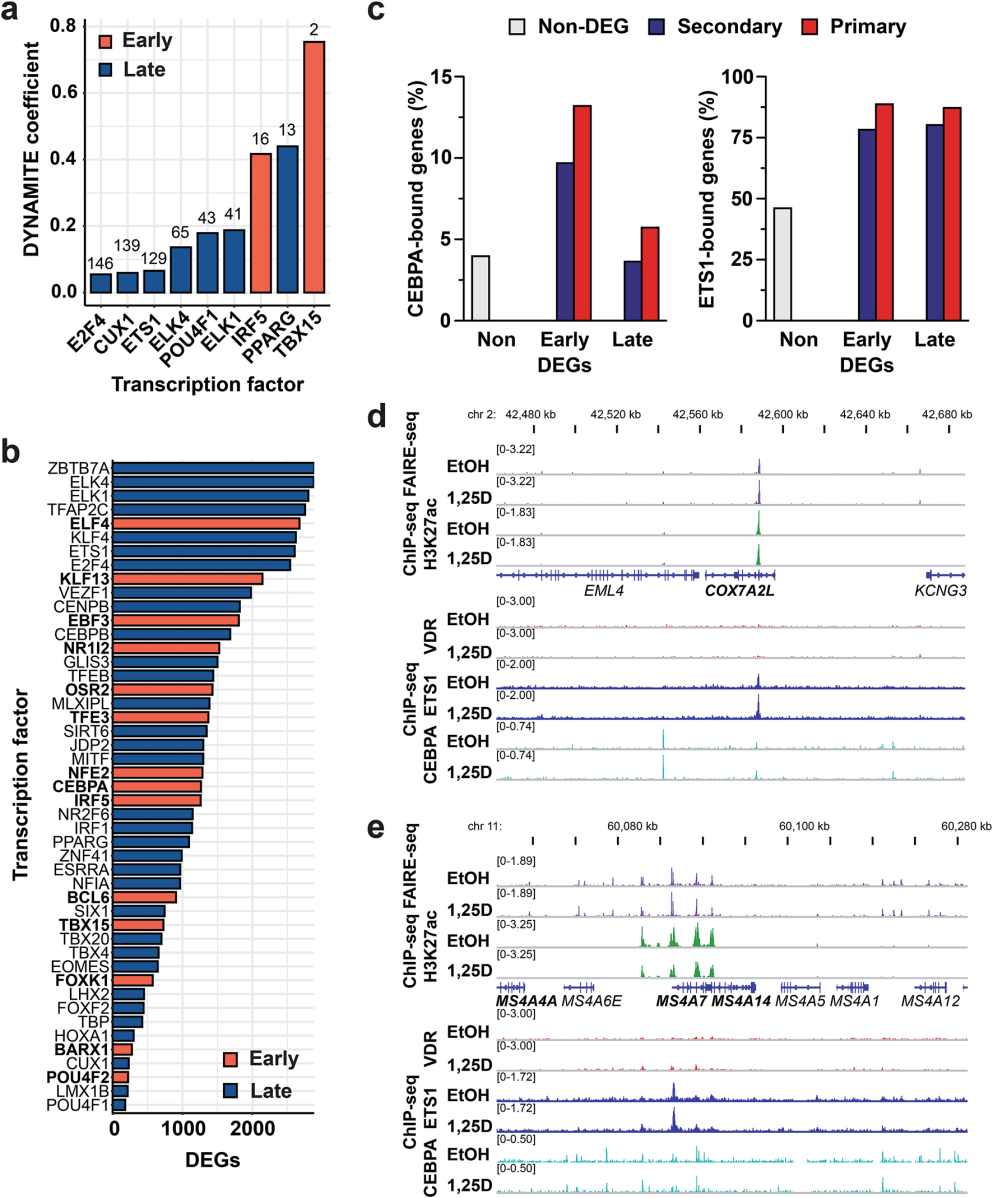
Analysis of primary VDR target transcription factors and their secondary vitamin D responses. The DYNAMITE analysis pipeline was used to identify primary VDR target transcription factors, which regulate secondary vitamin D target genes. Normalized absolute classifier coefficients from DYNAMITE are shown for primary VDR target transcription factors inside the top 150. On top of each bar the rank of each transcription factor in the entire DYNAMITE analysis is shown (**a**). The total numbers of DEGs after 24 h of 1,25(OH)_2_D_3_ treatment predicted to be targeted by each of the early and late induced primary 1,25(OH)_2_D_3_ target transcription factors comprised more than 2,000, including ETS1 or Kruppel like factor 4 (KLF4) (**b**). The ratio of early and late 1,25(OH)_2_D_3_-regulated genes to which CEBPA or ETS1 bind within + /- 25 kb of the TSS has been determined by ChIP-seq analyses (**c**). The predicted binding of ETS1 and CEBPA^13^ to the gene loci of the secondary VDR targets COX7A2L, *MS4A7* and *MS4A14* was confirmed by ChIP-seq assays, while no significant VDR association could be seen. The binding mostly occurred in open chromatin^15^ and co-located with the active enhancer mark H3K27ac^12^ (**d**, **e**). The gene names of all vitamin D target genes are shown in bold.

In order to better understand the regulation of secondary VDR target genes, the regulatory network of secondary transcription factors after 1,25(OH)_2_D_3_ treatment was characterized. Factors were prioritized that could explain secondary 1,25(OH)_2_D_3_-responsive genes, for which no direct VDR regulation could be established. For this purpose the DYNAMITE approach (see Methods) was used, in which motif based transcription factor binding prediction identifies meaningful transcription factors for a set of up- and down-regulated genes^46^. There were 22 transcription factors predicted to be of importance for the regulation of the secondary vitamin D target genes, amongst them *CUX1*, interferon regulatory factor 5 (*IRF5*)*, PPARG, NFE2* and *ETS1* (Fig. 4a and Supplementary Table S3).

In order to explain secondary effects of 1,25(OH)_2_D_3_, the TEPIC 2 framework was used to predict which of the late (24 h only) 1,25(OH)_2_D_3_-responsive genes are bound by the 47 primary vitamin D target transcription factors (Fig. 4b). Some transcription factors like ETS1 were predicted to associate to and potentially regulate more than 2,000 of the late vitamin D target genes. Obviously, many genes are regulated by more than one of these transcription factors. We chose to use the two pioneer transcription factors CEBPA and ETS1 to validate these predictions. For CEBPA, our previously published ChIP-seq dataset in 24 h ligand-stimulated THP-1 cells was used^13^, while for ETS1 a new duplicate ChIP-seq dataset in the same cell line was created. In solvent-treated cells, 20,838 ETS1 peaks were identified and 22,200 ETS-bound regions were present after treatment with the VDR ligand for 24 h, for each being present in both replicates. Interestingly, CEBPA preferentially bound close to the TSSs of early 1,25D-responding genes (+ /- 25 kb) and bound more often to primary than to secondary vitamin D target genes (Fig. 4c). ETS1 ChIP-seq peaks were observed at a much higher ratio of vitamin D targets, but there was no preference for early-responding genes and only a minor preference for primary target genes (Fig. 4c). CEBPA and ETS1 binding was among others predicted to associate to the gene loci of the secondary vitamin D target genes cytochrome c oxidase subunit 7A2 like (*COX7A2L*), membrane spanning 4-domains A14 (*MS4A14*), membrane spanning 4-domains A4A (*MS4A4*) and *LTK*. The ChIP-seq data confirmed strong ETS1 binding to a region close to the TSS of *COX7A2L* located in open chromatin and marked with the enhancer specific H3K27ac histone modification. CEBPA co-located to the same region and bound more strongly to another region in the locus (Fig. 4d). In the locus of the neighboring *MS4A14* and *MS4A4* genes, ETS1 was shown to bind to the TSS of *MS4A4* and CEBPA associated to several regions in the locus (Figs. 4e). Also in the *LTK* locus, CEBPA bound strongly to one region while several ETS1 binding sites were identified (Supplementary Figures S5a). The predicted CEBPA binding close to protein phosphatase 1 regulatory subunit 3G (*PPP1R3G*) was also confirmed by the CEBPA ChIP-seq data (Supplementary Figures S5b).

In order to determine whether the subsets of CEBPA or ETS1 bound vitamin D target genes represent different physiological functions, a pathway enrichment analysis was performed. No pathways were significantly connected to the vitamin D targets associated by CEBPA (Supplementary Table S4). The functional profile of the ETS1-targeted 1,25D-regulated genes comprised a broad range of different functions, the top hits being mainly metabolic pathways (Supplementary Table S4).

**Figure 5:**
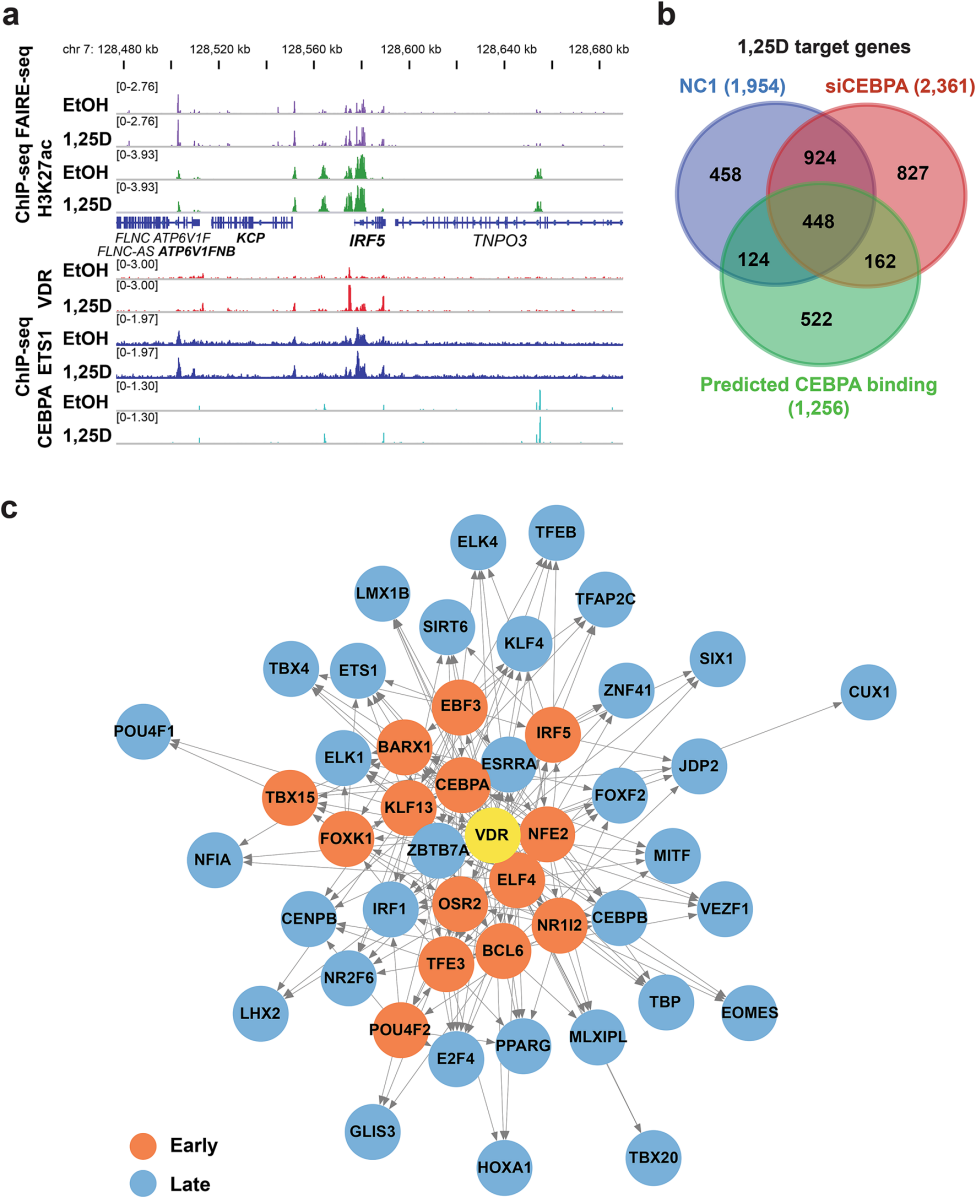
Higher level of regulation by secondary vitamin D responses on transcription factors. The predicted binding of VDR, ETS1 and CEBPA^13^ to the gene loci of the primary VDR target *IRF5* was confirmed by ChIP-seq assays. The binding occurred in open chromatin^15^ and co-located with the active enhancer mark H3K27ac^12^ (**a**). Please note that for the VDR ChIP-seq tracks^11^ the scale has been adapted to visualize medium-sized peaks, meaning that the summit of the strongest peak in both loci is cut. Intersects of the 1,25(OH)_2_D_3_ regulated genes identified from a previously published RNA-seq dataset in THP-1 cells with CEBPA knockdown^13^ with the predicted binding of CEBPA close to these genes (**b**). The directional network denotes which early primary VDR targeted transcription factors were predicted to bind at the gene loci of late primary VDR target transcription factors. All transcription factors shown were predicted to be bound at their gene loci by VDR (**c**).

TEPIC 2 analysis also showed that early, primary VDR targeted transcription factors could associate to the gene loci of late-induced primary 1,25(OH)_2_D_3_-regulated transcription factors. One example of this would be the predicted binding of VDR and the pioneer factors CEBPA and ETS1, whose mRNAs were significantly upregulated after 4 h of 1,25(OH)_2_D_3_ treatment, at the locus of the late 1,25(OH)_2_D_3_-induced *IRF5* gene. VDR associated strongly close to the TSS of the gene, to an intergenic enhancer and to an enhancer approximately 25 kb upstream of the TSS. CEBPA and ETS1 bound to at least two enhancers each (Fig. 5a). Likewise, the predicted binding of VDR, ETS1 and CEBPA close to the vitamin D target genes forkhead box K1 (*FOXK1*) and *ETS1* and the predicted binding of VDR and ETS1 to the gene coding for the general transcription factor TATA-box binding protein (TBP) were confirmed by the ChIP-seq data (Supplementary Table S6a-c).

A previously published RNA-seq dataset from THP-1 cells after knockdown of *CEBPA* verified that the predicted binding of CEBPA correlated with functional effects^13^. Of the vitamin D target genes identified from the wildtype THP-1 RNA-seq, 1,256 have been predicted to bind CEBPA. More than half of these (734) were also differentially expressed by 1,25(OH)_2_D_3_ in control or *CEBPA* knockdown cells, or both (Fig. 5b). Of these, 124 lost their response to 1,25(OH)_2_D_3_ by the knockdown, while 162 genes were differentially expressed by 1,25(OH)_2_D_3_ only in the absence of CEBPA. A rather high number of 448 vitamin D target genes were conserved vitamin D targets irrespective of the *CEBPA* silencing. However, as can be seen from the heatmap displaying the induction (fold change by 1,25(OH)_2_D_3_) of the top 50 of these genes, for most of them the *CEBPA* knockdown reduced the response to the VDR ligand (Figure S7a). In addition, the effects of 1,25(OH)_2_D_3_ treatment and CEBPA depletion is visualized for the exemplary transcription factor genes *NFIA*, transcription factor binding to IGHM enhancer 3 (*TFE3*) and GLIS family zinc finger 3 (*GLIS3*) (Figures S7b-d).

The interrelation of all transcription factors that were differentially expressed by long or short 1,25(OH)_2_D_3_ stimulation, were directly targeted by VDR, and have been predicted to also regulate other transcription factors, is summarized in a VDR-centered directional regulatory network (Fig. 5c).

In summary, CEBPA and ETS1 ChIP-seq data validated the predicted association of these transcription factors to secondary vitamin D target genes. Approximately half of the vitamin D target genes predicted to bind CEBPA showed lost, gained or modulated response to 1,25(OH)_2_D_3_ following CEBPA depletion. A directional network containing 47 primary VDR target transcription factors indicated a high complexity of vitamin D signaling and suggests novel factors.

## Discussion

In this study, we used a hierarchical analysis of the 1,25(OH)_2_D_3_-regulated transcriptome in monocytic THP-1 cells to predict primary VDR target genes on the basis of RNA-seq, VDR ChIP-seq, FAIRE-seq, VDR motif analysis and the EpiRegio database of REMs. Our analysis indicated that the classification of 1,25(OH)_2_D_3_ DEGs into primary and secondary vitamin D target genes just by time points is an oversimplification. A high proportion (72.1%) of all early (2.5 or 4 h) 1,25(OH)_2_D_3_-regulated genes were predicted to be direct VDR targets, which may not seem surprising as it fits well with the time point-based classification. However, from the late-responding genes (24 h), 56.3% were assigned primary targets by our approach, which is a high ratio considering that these genes are usually considered secondary target genes due to their slow response. From the three approaches we applied, VDR ChIP-seq peaks were assigned to the highest numbers of vitamin D-responsive genes. Among them were many genes for which a VDR binding site was not predicted by TEPIC 2. This is likely due to imperfect motif models of the complex VDR binding profile, or the binding of VDR with co-factors resulting in the presence of VDR in regions where no canonical VDR binding motif is present. It is also in line with the observation that only 47.6% of the VDR cistrome carried a DR3-type VDR motif^11^.

Increasing the stringency by classifying only those DEGs as primary, which have been predicted by at least two of the three prediction methods reduced the ratio of primary target genes to 45.6% and 24.4% for early and late responding genes, respectively. Thus, this would indicate that 54.4% of the early regulated genes might in fact be secondary vitamin D target genes. The stringency of the prediction methods greatly depends on the window sizes that have been applied. For the FAIRE-seq motif and ChIP-seq approaches performed in TEPIC 2, we used a window of ± 25 kb around TSSs of the genes. Whilst we were aware that enhancers could be more distal from target genes than the threshold suggested by that window size, an increased window size would have led to many more false positive primary target gene predictions. On the other hand, it could be argued that none of the early 1,25(OH)_2_D_3_-responding genes can be a secondary target gene and thus our predictions have been too stringent. Inducible transcription factors lead to bursts of transcription and the bursting period commonly lasts several hours during which several transcriptional events take place^71–75^. For a secondary response, after the several hours of primary transcriptional activation the transcription factor has to be translated and then may induce a second transcriptional activation on its own. Thus, it is very unlikely that secondary responses can be detected in the time frame of 4 h. However, no matter which stringency we apply, a major outcome of this study is that a significant number of all DEGs that are unique to the 24 h time point of 1,25(OH)_2_D_3_ stimulation are primary VDR target genes. There is some inaccuracy in all RNA-seq data, which we have observed for example for the *CXCL8* (*IL8*) gene. According to both RNA-seq datasets used in this study, the gene is not significantly regulated after 2.5 or 4 h stimulation with 1,25(OH)_2_D_3_ and was therefore classified as late response gene. However, in a previous report the gene has been shown to be significantly upregulated by 1,25(OH)_2_D_3_ in THP-1 cells already after 1 h by RT-qPCR^76^. If the same were true for many of the late responding genes, the discordance between primary target gene predictions and time point-based classification would be reduced. A gene may be directly regulated by VDR, but mRNA accumulation may be low if either the transcriptional burst is small or if mRNA stability is low. In such cases, the detection by RNA-seq may only be possible with very high sequencing depth. Assuming that not only technical reasons prevented the detection of weak mRNA levels accurately, our data indicate that for some primary vitamin D target genes the transcriptional response is delayed. This could be due to differences in co-activator recruitment or co-repressor disposal or to lower local chromatin accessibility. Also, the number and position of VDR sites in the gene locus may play a role. It has to be noted, though, that there is a rather big gap between the 4 h and the 24 h time-point and some genes may already be significantly regulated after 6 h or 8 h and thus may not be that late-responding after all. In contrast to the TEPIC 2-based approaches, the REMs in the EpiRegio database have been determined in a window of + /100 kb around and including the gene body. In spite of that less stringent window size, there are rather few primary target gene predictions made by this approach that have not been confirmed by at least one of the other two approaches. The REMs have been determined from large numbers of genome-wide datasets, which highly increased the statistical power of this method and rendered it more quantitative.

Knockout of the *VDR* gene completely abolished 1,25(OH)_2_D_3_-induced transcriptomic changes as measured by RNA-seq. This on the one hand validated the identified vitamin D target genes, which could no longer be regulated by 1,25(OH)_2_D_3_ if its nuclear receptor VDR was not present to act as a ligand-activated transcription factor. It also indicated that even weakly up- or downregulated genes are indeed true vitamin D target genes and presumably their regulation plays a biological role. On the other hand, in light of reports about non-genomic effects of 1,25(OH)_2_D_3_ which have been described to also alter the activity of transcription factors like RXR and thus crosstalk with transcriptional regulation by its heterodimerization partner VDR^41,44^, it is surprising that there are no genes differentially expressed by 1,25(OH)_2_D_3_ in the *VDR* knockout cells. This contradicts reports about such rapid 1,25(OH)_2_D_3_ actions being independently of VDR mediated by an alternative receptor as PDIA3^18,31–39^ and indicates that in the monocytic THP-1 cells such rapid effects of 1,25(OH)_2_D_3_ either involve the classical VDR by binding to its alternative binding pocket^28,29^, or are not present at all.

Another major result of this study is that there are 47 transcription factors whose expression was regulated by 1,25(OH)_2_D_3_ and which were predicted to be responsible for secondary effects of the active form of vitamin D. Each of these transcription factors was predicted to bind at many vitamin D-responsive genes, with some linked to thousands of DEGs. Among these were primary and secondary target genes, some of these being transcription factors as well. Using ChIP-seq data for the pioneer factors ETS1 and CEBPA, we could validate some of the predicted associations to vitamin D target gene loci. The myeloid lineage-determining transcription factor CEBPA is known to co-locate with VDR at more than 5,000 regions and, as an additional layer of complexity, at ≈1,500 sites CEBPA binding was modulated by the VDR ligand^13^. *CEBPA* silencing affected both the basal expression levels and the induction by 1,25(OH)_2_D_3_ of more than half of all strongly regulated vitamin D target genes. The latter represents both the pioneering function of the transcription factor, where CEBPA co-locates with VDR at primary VDR target genes, and the regulation of genes by CEBPA that are no direct VDR targets. This regulation was increased by the 1,25(OH)_2_D_3_-induced upregulation of *CEBPA* expression and by increased binding of CEBPA to some of its binding sites. We could correlate the predicted binding of CEBPA to a few hundred vitamin D target genes to functional changes by the CEBPA knockdown. This confirmed about a quarter (based on significant changes of the vitamin D response) to half (including non-significant changes) of our predictions for this transcription factor. There are additionally more than 1,000 genes that either lost or gained response to 1,25(OH)_2_D_3_ when depleting CEBPA, but were not predicted to bind CEBPA. These genes may represent secondary CEBPA target genes, or they were not predicted as CEBPA-bound due to imperfect CEBPA motif models or indirect DNA-association of CEBPA in a complex with other transcription factors. Previously, *VDR* and *CEBPA* have been shown to be tightly co-expressed in a module that is up-regulated in monocytes compared to cells from other hematopoietic lineages^77^. According to that study many transcription factors can be assembled into densely interconnected transcriptional circuits, which can provide a mechanism for robust gene regulation. Our results are inline with these findings and may be an explanation why monocytic cells are much more responsive to 1,25(OH)_2_D_3_ than some epithelial cell-types even though VDR expression is similarly high in both. Most probably other modules are highly expressed in these epithelial cells and if certain co-activators, chromatin-modifiers or vitamin D-regulated transcription factors are not in the expressed modules together with *VDR* the responsiveness to the VDR ligand will be lower. We presented a VDR-centered directional network of 47 VDR targeted transcription factors to describe the complexity of global vitamin D signaling in THP-1 cells. Most probably this network is cell-type specific and will contain partly other transcription factors in other cells. The often very strong increase in mRNA expression levels of primary vitamin D target genes from short to long time-points of ligand stimulation can partly be explained by such additional secondary 1,25(OH)_2_D_3_ responses.

In summary, we applied a novel hierarchical approach to distinguish primary from secondary 1,25(OH)_2_D_3_ actions and to mechanistically characterize secondary effects of the VDR ligand in THP-1 cells. We predicted a high proportion of late-responding DEGs, that commonly are considered secondary target genes, to be in fact primary VDR targets. The remaining secondary VDR target genes appear to be regulated by a network of 47 transcription factors whose expression is directly regulated by 1,25(OH)_2_D_3_. This network is VDR-centered as we could show that all transcriptome-wide effects of the VDR ligand depended strictly on the presence of VDR. Thus, any non-genomic effects of 1,25(OH)_2_D_3_ that may modulate its transcriptional function, have to be mediated by VDR as well. The main findings from this study refined our previously published model of vitamin D signaling^78^ of which an adapted version is depicted in Supplementary Figure S8.

## Supplementary Information

Supplementary files.

Supplementary Table 2

Supplementary Table 3

Supplementary Table 4

## Data Availability

The ETS1 ChIP-seq data are available at GEO at GSE157209 and the VDR knockout RNA-seq at GEO157514.

## Abbreviations

1,25(OH)_2_D_3_ or 1,25D: 1α,25-Dihydroxyvitamin D_3_
1,25D3-MARRS: Membrane associated rapid response steroid binding
BCL6: B-cell CLL/lymphoma 6
CAMP: Cathelicidin antimicrobial peptide
Cas9: Type II CRISPR RNA-guided endonuclease Cas9
CEBPA: CCAAT/enhancer binding protein α
ChIP: Chromatin immunoprecipitation
ChIP-seq: ChIP sequencing
COX7A2L: Cytochrome c oxidase subunit 7A2 like
CUX1: Cut like homeobox 1
CXCL8: C-X-C motif chemokine ligand 8 (interleukin 8)
CRISPR: Clustered regularly interspaced short palindromic repeats
DEG: Differentially expressed gene
DR3: Direct repeat spaced by 3 nucleotides
ELF4: E74 like ETS transcription factor 4
ETS1: ETS proto-oncogene 1, transcription factor
FAIRE-seq: Formaldehyde-assisted isolation of regulatory elements sequencing
FCS: Fetal calf serum
FOXK1: Forkhead box K1
GEO: Gene Expression Omnibus
GLIS3: GLIS family zinc finger 3
gRNA: Guide RNA
HBEGF: Heparin binding EGF like growth factor
HOCOMOCO: HOmo sapiens COmprehensive MOdel COllection
IGV: Integrative Genomics Viewer
IRF5: Interferon regulatory factor 5
KCNF1: Potassium voltage-gated channel modifier subfamily F member 1
KLF4: Kruppel like factor 4
KO: Knockout
LTK: Leukocyte receptor tyrosine kinase
MACS: Model-based Analysis of ChIP-Seq data
MAPK: Mitogen-activated protein kinase
MS4A4/14: Membrane spanning 4-domains A4/14
NFE2: Nuclear factor erythroid 2
NFIA: Nuclear factor I A
NINJ1: Ninjurin 1
PBS: Phosphate-buffered saline
PDIA3: Protein disulphide isomers family A member 3
PEI: Polyethylenimine
PI3K: Phosphatidylinositol 3-kinase
PKC: Proteinkinase C
PKCaMII: Ca2 + /calmodulin-dependent protein kinase
PLA2: Phospholipase A2
PLC: Phospholipase C
POU4F2: POU class 4 homeobox 2
PPARG: Peroxisome proliferator activated receptor gamma
PPP1R3G: Protein phosphatase 1 regulatory subunit 3G
PU.1: Purine-rich box 1, Spi-1 proto-oncogene (official gene symbol: *SPI1*)
REM: Regulatory element
RNA-seq: RNA sequencing
RXR: Retinoid X receptor
STAR: Spliced Transcripts Alignment to a Reference
TBP: TATA-box binding protein
TFE3: Transcription factor binding to IGHM enhancer 3
TSS: Transcription start site
VDR: Vitamin D receptor
VSV-G: Glycoprotein of vesicular stomatitis virus
ZFP36: ZFP36 ring finger protein
