# Supplementary material for: A hierarchical regulatory network analysis of the vitamin D induced transcriptome reveals novel regulators and complete VDR dependency in monocytes
Source: Sci Rep. 2021 Mar 22;11:6518. doi: 10.1038/s41598-021-86032-5 (PMC7985518; doi:10.1038/s41598-021-86032-5)
Supplement: Supplementary file 1 — Supplementary files. [file 41598_2021_86032_MOESM1_ESM.pdf]

A hierarchical regulatory network analysis of the vitamin D induced transcriptome reveals novel regulators and complete VDR dependency in monocytes

Timothy Warwick[a,c], Marcel H. Schulz[b,c], Stefan Günther[d], Ralf Gilsbach[a,c], Antonio Neme[e,f], Carsten Carlberg[e], Ralf P. Brandes[a,c] and Sabine Seuter[a,c,e#]

[a] Institute for Cardiovascular Physiology, Goethe University, Frankfurt/Main, Germany

[b] Institute for Cardiovascular Regeneration, Goethe University, Frankfurt/Main, Germany

[c] German Center for Cardiovascular Research (DZHK), Partner site Rhein-Main, 60590 Frankfurt am Main, Germany

[d] Max-Planck-Institute for Heart and Lung Research, Bad Nauheim, Germany

[e] School of Medicine, Institute of Biomedicine, University of Eastern Finland, FI-70211 Kuopio, Finland

[f] Institute for Applied Mathematics, Merida Research Unit, National Autonomous University of Mexico, Sierra Papacal Merida Yucatan, C.P. 97302, Mexico

Fig. S1

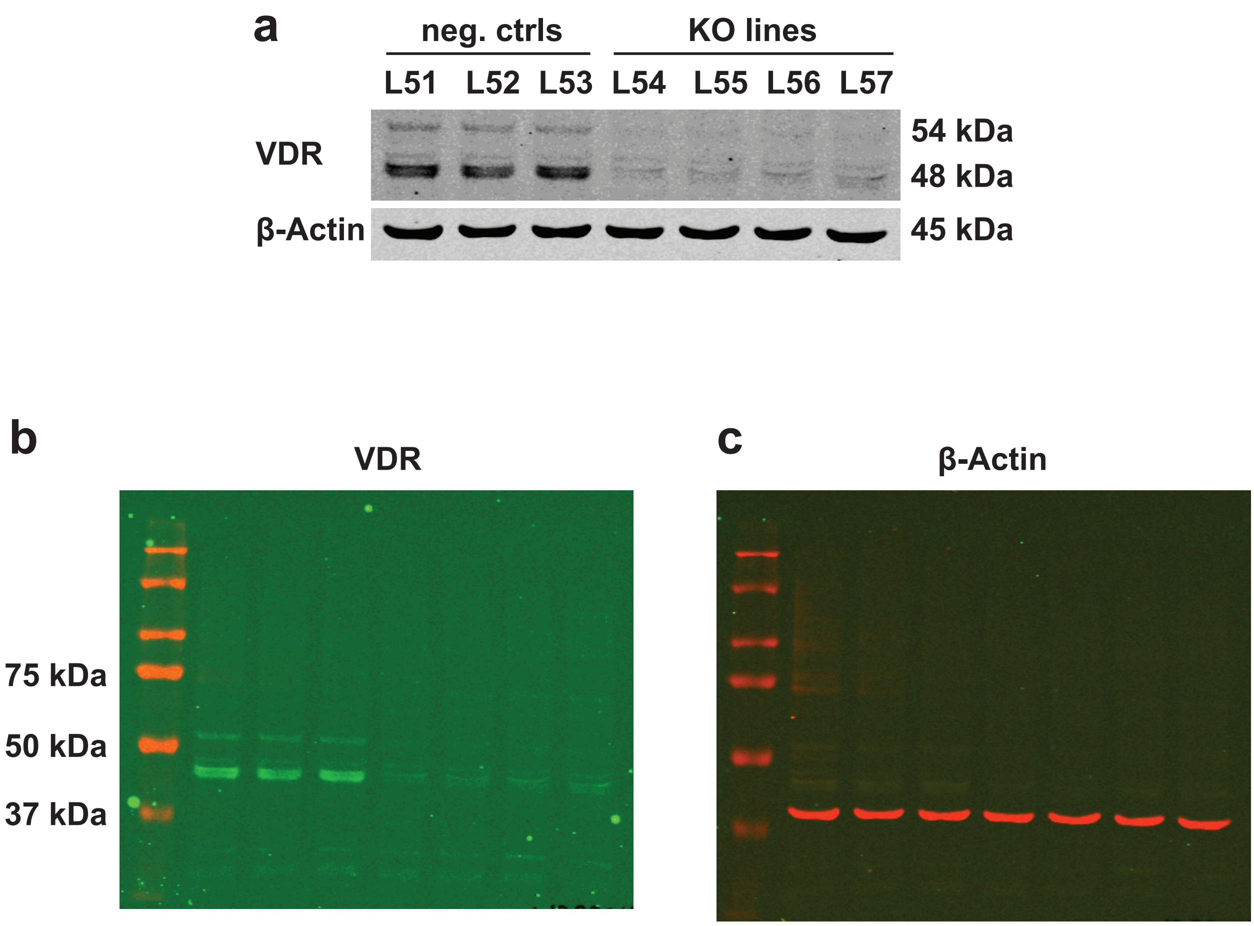

Figure S1: VDR knockout. The successful knockout of the VDR protein by CRISPR/Cas9 genome editing was verified by Western Blot in four different knockout lines (L54-L57), created by lentiviral transduction of four different gRNAs (Supplementary Table S1), compared to three different controls (L51 and L52 – non-targeting gRNAs NTC1 and NTC2, L53 – empty vector). The assembled bands converted to greyscale are shown in **a**, while the complete original scans of the blot are depicted in **b** (VDR) and **c** (β-Actin). The Precision Plus Protein All Blue Prestained Protein Standards (BioRad) were loaded as size marker. VDR knockout line L56 and control line L52 were used in the subsequent experiments.

Fig. S2

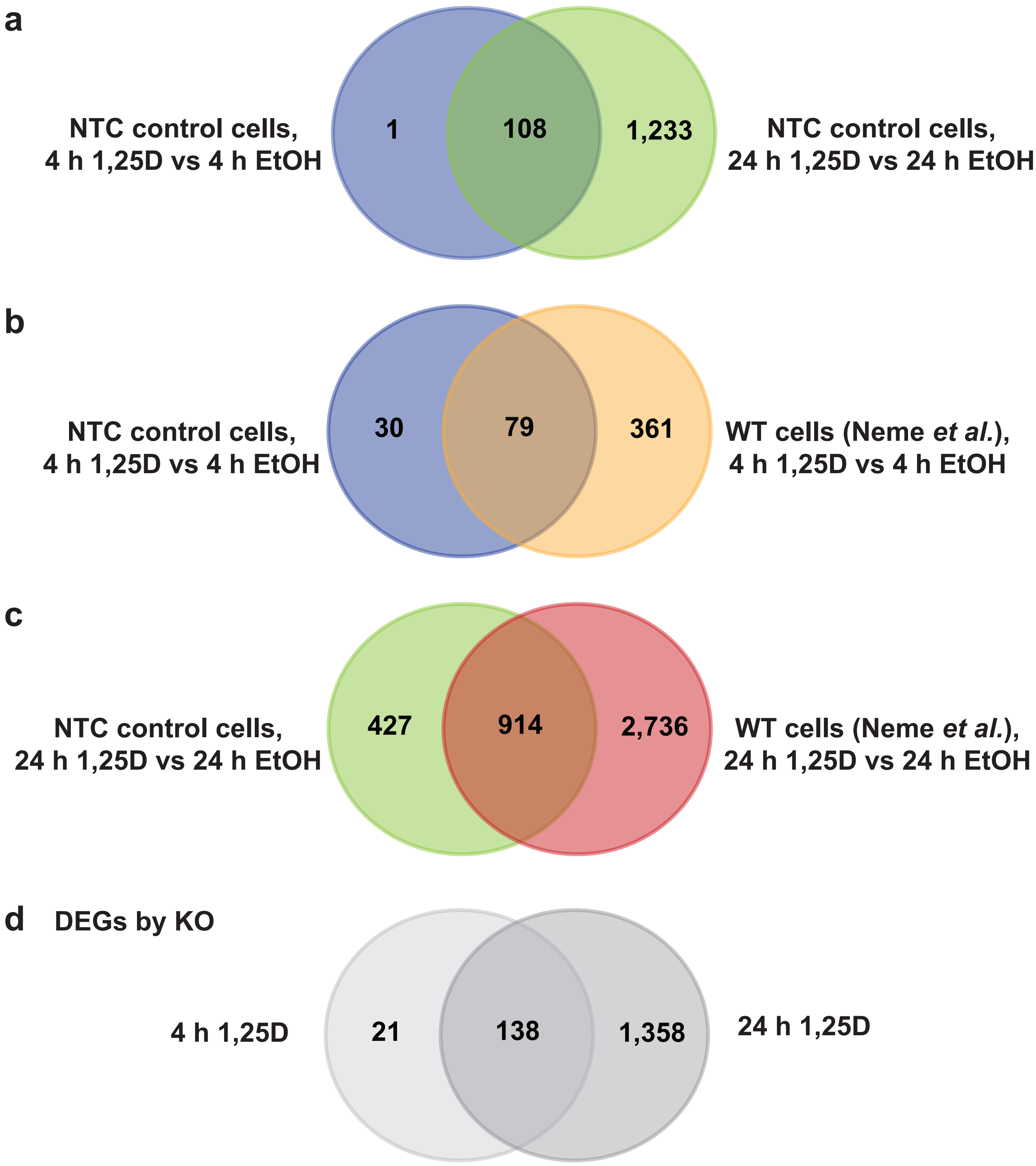

Figure S2: **Differential analysis of the 1,25(OH)$_2$D$_3$-regulated transcriptome in CRISPR/Cas9 control cells.** THP-1 cells that have been transduced with a non-targeting gRNA (NTC) have been treated with 1,25(OH)$_2$D$_3$ or solvent (EtOH) for 4 or 24 h and four biological repeats of RNA-seq have been performed. The huge intersect between both time points (a) as well as the reasonably high overlap with the DEGs that had recently been identified by RNA-seq from THP-1 wild-type cells (b, c) indicate consistency with our previous studies. There was a strong increase in the number of genes differentially expressed by the VDR knockout in 24 h 1,25(OH)$_2$D$_3$ treated cells compared to 4 h stimulated cells and the majority of the early time point DEGs overlap with those of the late time point (d).

Fig. S3

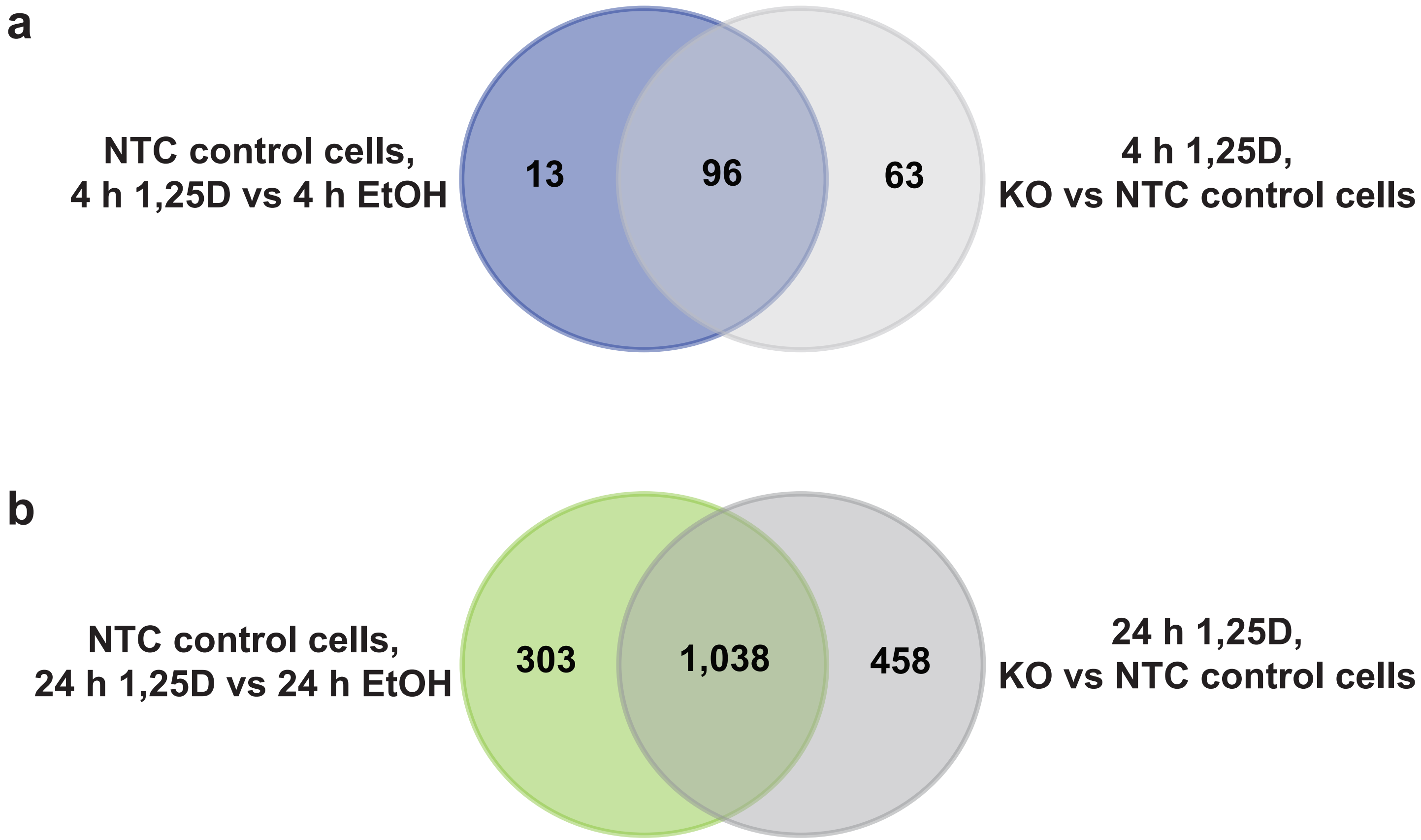

Figure S3: High overlap between 1,25(OH)$_2$D$_3$-regulated genes and genes with modulated expression by the absence of VDR. THP-1 cells that have been transduced with a VDR-specific (KO) or a non-targeting gRNA (NTC) have been treated with 1,25(OH)$_2$D$_3$ or solvent (EtOH) for 4 or 24 h and quadruplicate RNA-seq has been performed. Intersects between vitamin D target genes and genes differentially expressed by the VDR knockout for 4 h (a) and 24 h (b) VDR ligand treatment.

Fig. S4

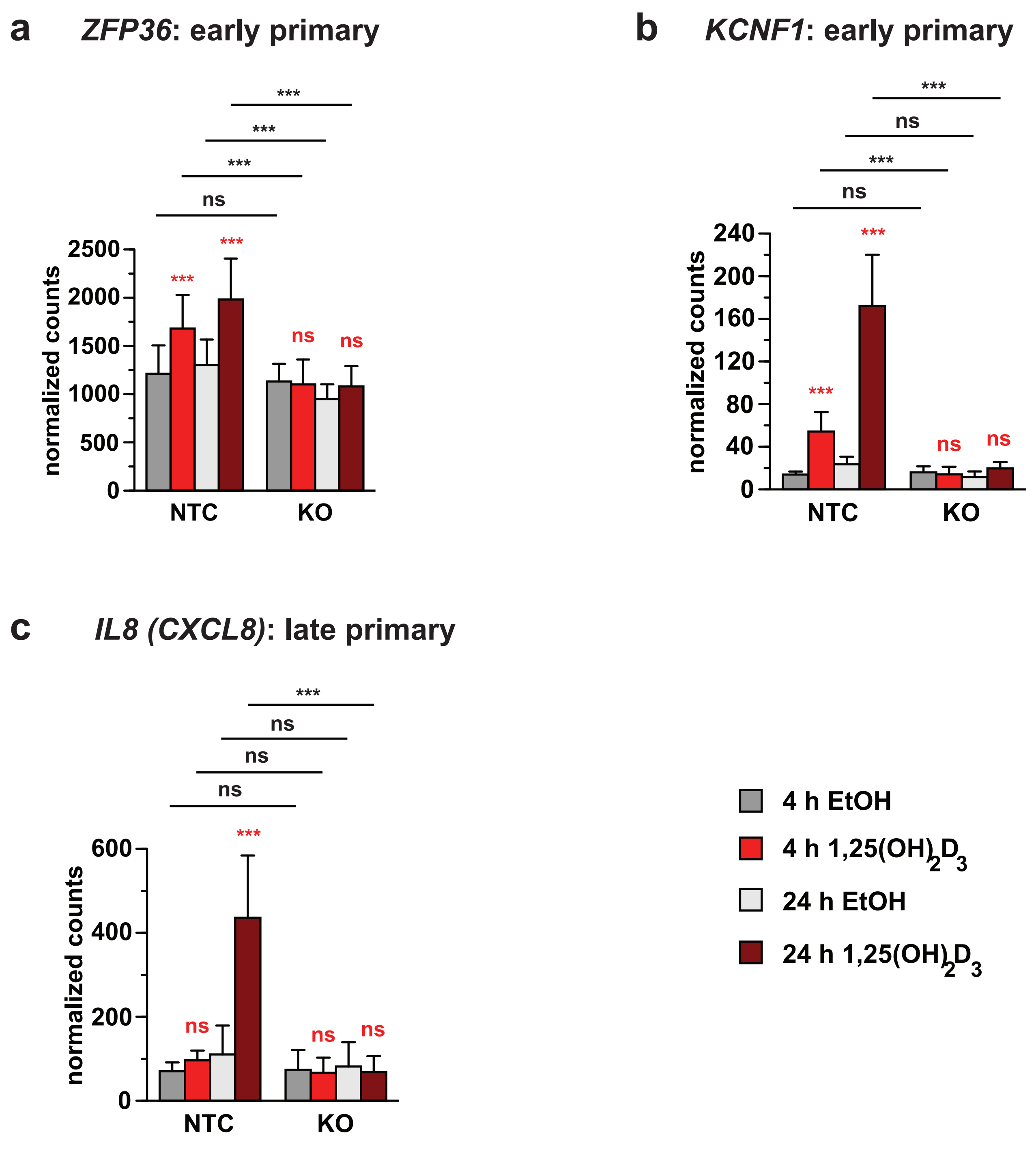

Figure S4: VDR knockout abolished transcriptomic vitamin D response. THP-1 cells that have been transduced with a VDR-specific (KO) or a non-targeting gRNA (NTC) were treated with 1,25(OH)$_2$D$_3$ or solvent (EtOH) for 4 or 24 h and quadruplicate RNA-seq was performed. The effects of the VDR knockout are displayed for two early primary (a, b) and one late primary (c) vitamin D target genes.

Fig. S5

a

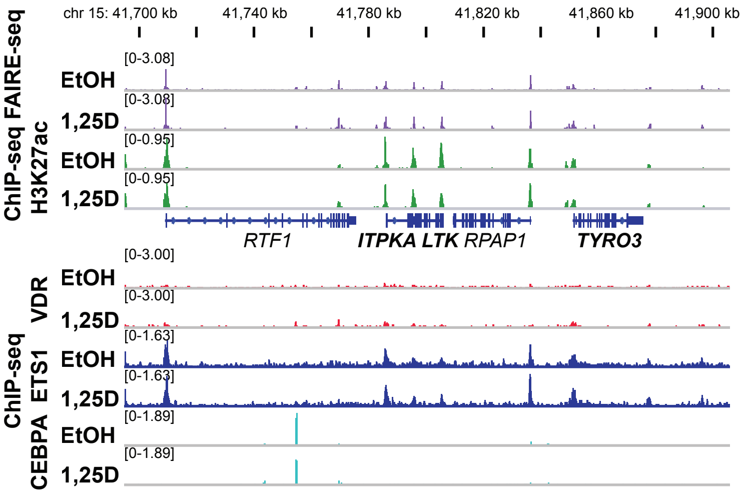

b

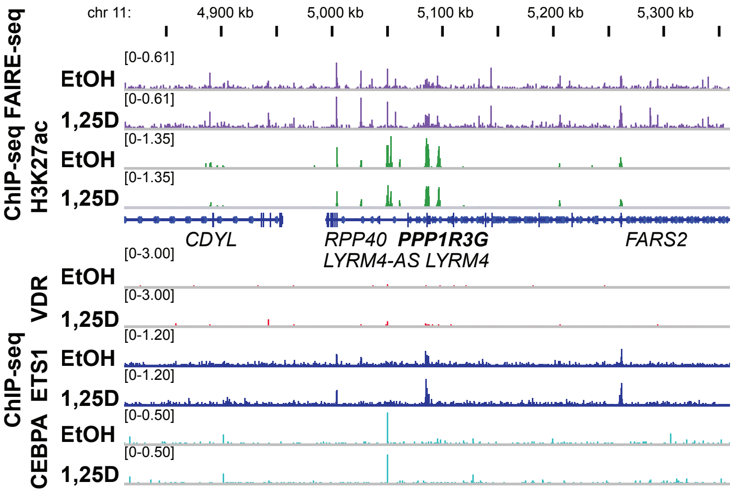

Figure S5: Transcription factor binding to the locus of secondary 1,25(OH)$_2$D$_3$-regulated genes. The predicted binding of CEBPA and ETS1 to the gene locus of the secondary VDR targets *LTK* (a) and of CEBPA to the *PPP1R3G* locus (b) was confirmed by ChIP-seq assays, while no significant VDR association could be seen. The binding occurred in open chromatin and co-located with the active enhancer mark H3K27ac. The gene names of all vitamin D target genes are shown in bold.

Fig. S6

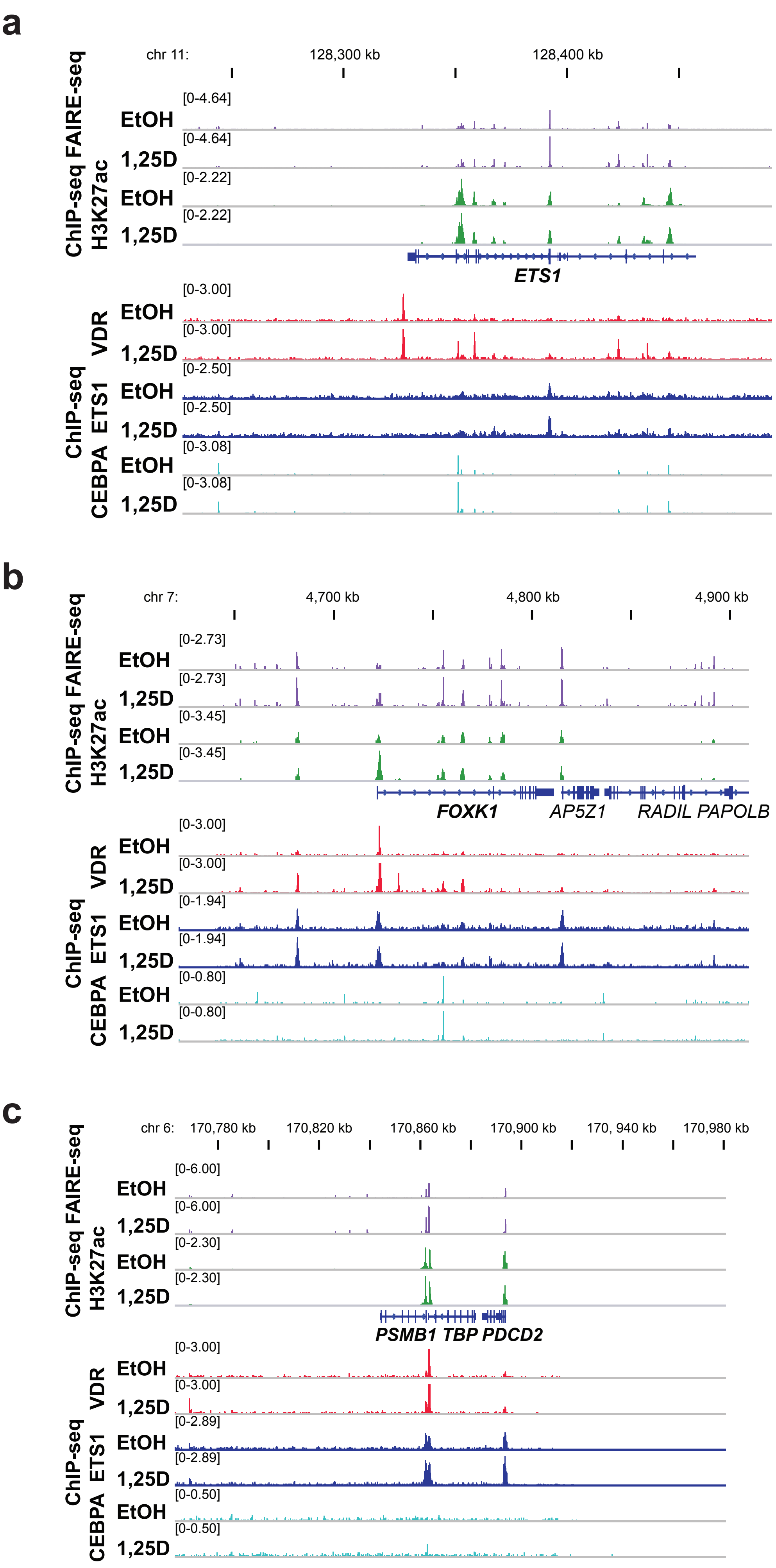

Figure S6: Transcription factor binding to loci of primary VDR target transcription factors. The predicted binding of VDR, ETS1 and CEBPA to the gene locus of the primary VDR target genes *ETS1* and *FOXK1* (a, b) and of VDR and ETS1 to *TBP* (c) was confirmed by ChIP-seq assays. The binding occurred in open chromatin and co-located with the active enhancer mark H3K27ac. Please note that for the VDR ChIP-seq tracks the scale has been chosen such as to be able to see also medium-sized peaks, meaning that the summits of the strongest peaks are cut. The gene names of all vitamin D target genes are shown in bold.

Fig. S7

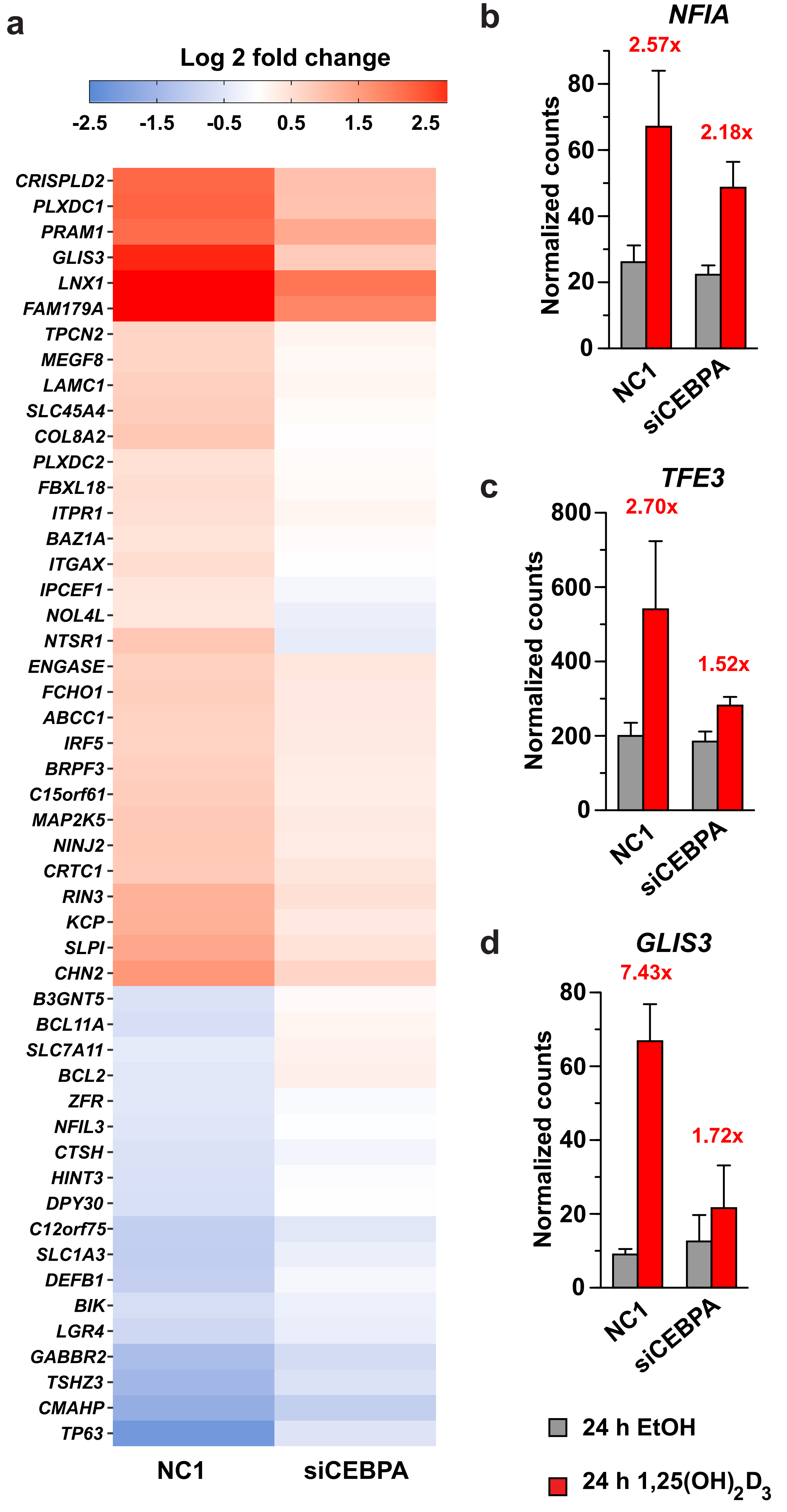

Figure S7: Effects of CEBPA knockdown on vitamin D target genes, to which CEBPA was predicted to bind. From the $1,25(OH)_2D_3$ regulated genes identified from a previously published RNA-seq dataset in THP-1 cells with CEBPA knockdown that were predicted to bind CEBPA we selected those that do not gain or lose their response to the VDR ligand by the CEBPA knockdown (Figure 5). The log2 fold changes in gene expression of the top 50 most significant of these DEGs in both siCEBPA and NC1 control cells demonstrated the almost complete loss of $1,25(OH)_2D_3$ response (a). The genes *NFIA*, *TFE3* and *GLIS3* are shown to further illustrate this effect (b).

Fig. S8

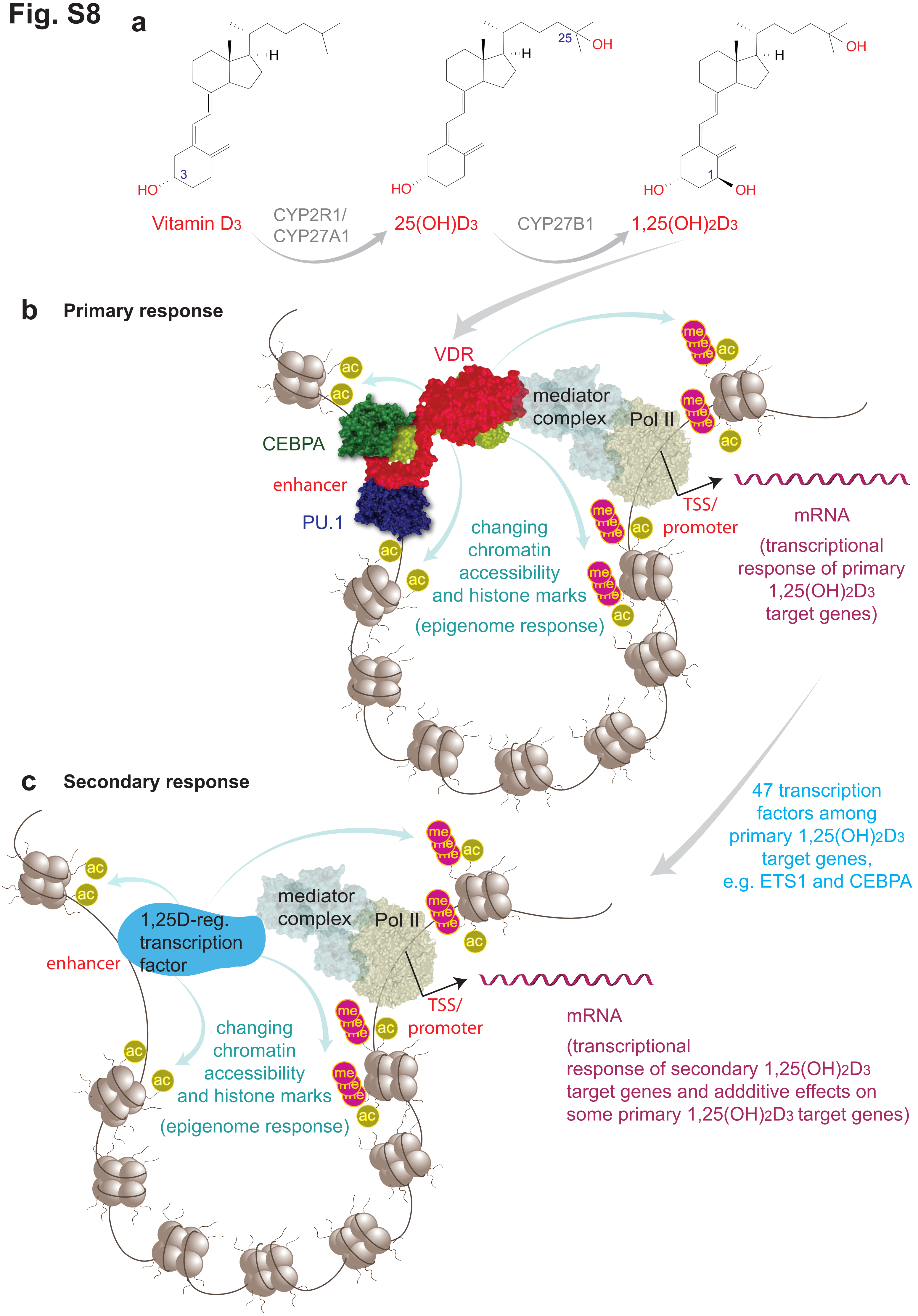
a
3
HO
OH
H
25
1
Vitamin D3
CYP2R1/
CYP27A1
25(OH)D3
CYP27B1
1,25(OH)2D3
b
Primary response
VDR
ac
me
mediator
complex
Pol II
CEBPA
enhancer
PU.1
changing
chromatin
accessibility
and histone marks
(epigenome response)
TSS/
promoter
mRNA
(transcriptional
response of primary
1,25(OH)2D3
target genes)
c
Secondary response
47 transcription
factors among
primary 1,25(OH)2D3
target genes,
e.g. ETS1 and CEBPA
1,25D-reg.
transcription
factor
enhancer
mediator
complex
Pol II
TSS/
promoter
changing
chromatin
accessibility
and histone marks
(epigenome response)
mRNA
(transcriptional
response of secondary 1,25(OH)2D3
target genes and addditive effects on
some primary 1,25(OH)2D3 target genes)

Figure S8: Model of vitamin D signaling. Vitamin D_3 is hydroxylated to 25-hydroxyvitamin D_3 (25(OH)D_3) and the high affinity VDR ligand 1,25$(OH)_2D_3$ (a). Pioneer transcription factors, such as PU.1 (dark blue) and CEBPA (green), increase chromatin accessibility and facilitate binding of VDR to enhancer sites and histone acetylation (H3K27ac) (b). Interaction of VDR-bound enhancers with mediator complexes at TSS regions induces changes of histone marks at promoters (H3K4me3 and H3K27ac) and activates RNA polymerase II. Subsequently, the respective primary vitamin D target genes are transcribed. Among these primary vitamin D targets are 47 transcription factor genes. When these transcription factors are translated, they associate to their binding sites at enhancer regions to regulate genes which are secondary vitamin D target genes or primary target genes that are regulated by the other transcription factor as well (c).

SUPPLEMENTARY DATA

SUPPLEMENTARY TABLES

Table S1: Oligonucleotides for gRNA cloning into lentiGuide-Puro (Addgene #52963).

Gene	Position in chr12 (hg19)	Oligonucleotide sequences (5'-3')	KO line
VDR	47,857,227	CACCGCATCATTCACACGAACTGG<br>AAACCCAGTTCGTGTGAATGATGC	L57
VDR	47,857,532	CACCGGGGTCGTAGGTCTTATGGT<br>AAACACCATAAGACCTACGACCCC	L56
VDR	47,865,123	CACCGGCGGCAGTCCCCGTTGAAG<br>AAACCTTCAACGGGGACTGCCGCC	L55
VDR	47,879,056	CACCGCGTTCCGGTCAAAGTCTCC<br>AAACGGAGACTTTGACCGGAACGC	L54
NTC1	non-targeting control	CACCGTTCCGGGCTAACAAGTCCT<br>AAACAGGACTTGTTAGCCCGGAAC	L51
NTC2	non-targeting control	CACCGAATATCAACGCGAGCAAGG<br>AAACCCTTGCTCGCGTTGATATTC	L52

Table S2: Vitamin D-triggered transcriptome of VDR knockout and control cells. The VDR was knocked out by CRISPR/Cas9 genome editing in THP-1 cells. VDR knockout (KO) cells and control (NTC) cells were treated with $1,25(OH)_2D_3$ (V) or solvent (EtOH; E) for 4 or 24 h and four biological repeats (N1-N4) of RNA-seq have been performed. The raw data were analyzed using STAR for genomic alignment and DESeq2 for differential expression for different pairwise comparisons between the conditions.

Table S3: Prediction of primary VDR target transcription factors to mediate secondary vitamin D responses. The DYNAMITE analysis pipeline was used to identify primary VDR targeted transcription factors (TFs) with importance for the regulation of secondary vitamin D target genes. Normalized absolute classifier coefficients are listed alongside the rank in the entire DYNAMITE analysis for primary VDR targeted transcription factors**.**

Table S4: Pathway enrichment analysis for the 1,25(OH)$_2$D$_3$-regulated genes targeted by CEBPA or ETS1. ReactomePA was used to identify pathways that are enriched in the DEG subsets that have been shown to be bound by CEBPA or ETS1 within -/-25 kb of their TSS by ChIP-seq. BgRatio, background ratio; GeneRatio, number of genes of interest annotated to the pathway, out of the total size of the geneset of interest.
